# Immunological risk factors for nonalcoholic fatty liver disease in patients with psoriatic arthritis: New predictive nomograms and natural killer cells

**DOI:** 10.3389/fimmu.2022.907729

**Published:** 2022-07-22

**Authors:** Baochen Li, Rui Su, Huanhuan Yan, Juanjuan Liu, Chong Gao, Xiaofeng Li, Caihong Wang

**Affiliations:** ^1^ Department of Rheumatology, the Second Hospital of Shanxi Medical University, Taiyuan, China; ^2^ Department of General Medicine, the Second Hospital of Shanxi Medical University, Taiyuan, China; ^3^ Department of Pathology, Brigham and Women’s Hospital, Harvard Medical School, Boston, MA, United States

**Keywords:** psoriatic arthritis, nonalcoholic fatty liver disease, nomogram, dyslipidemia, T helper-1 cells, natural killer cells

## Abstract

**Objective:**

To search for the immunological risk factors of Psoriatic arthritis (PsA) combined with nonalcoholic fatty liver disease (NAFLD), development and assessment of predictive nomograms for NAFLD risk in patients with PsA, and to further explore the correlation between risk factors and dyslipidemia.

**Methds:**

A total of 127 patients with PsA (46 with NAFLD and 81 without NAFLD) were included in this retrospective study. The clinical and serological parameters of the patients were collected. The percentage and the absolute number of lymphocytes and CD4+T cells were determined by Flow cytometry. Univariate and multivariate binary logistic regression analysis was used to screen independent risk factors of PsA complicated with NAFLD in the model population, and a nomogram prediction model was developed and assessed.

**Results:**

(1) Univariate and multivariate logistic regression analysis of the modeling population showed that the percentage of peripheral blood T helper 1 cells (Th1%) (OR=1.12, P=0.001), body mass index (BMI) (OR=1.22, P=0.005) and triglycerides (TG) (OR=4.78, P=0.003) were independent risk factors for NAFLD in patients with PsA, which were incorporated and established a nomogram prediction model. The model has good discrimination and calibration, and also has certain clinical application value. (2) The number of peripheral blood NK cells in PsA patients was significantly positively correlated with serum triglyceride (TG) (r=0.489, P<0.001), cholesterol (CHOL) (r=0.314, P=0.003) and low-density lipoprotein (LDL) (r=0.362, P=0.001) levels.

**Conclusions:**

Our study shows that the novel NAFLD nomogram could assess the risk of NAFLD in PsA patients with good efficiency. In addition, peripheral blood NK cell levels may be associated with dyslipidemia in patients with PsA.

## Introduction

Psoriatic arthritis (PsA) is a chronic, immune-mediated inflammatory joint disease characterized by progressive inflammatory destruction of the axial and peripheral joints. In recent years, the inflammatory process of the pathophysiological changes of PsA has become the focus of attention. Chronic low-grade systemic inflammation associated with skin inflammation is thought to underlie many comorbidities of PsA, including cardiovascular disease, metabolic syndrome, and nonalcoholic fatty liver disease (NAFLD) (1).

NAFLD is a liver disease characterized by hepatic steatosis, with a global incidence of about 25%, and is currently one of the leading causes of liver cirrhosis and liver transplantation (2). A recent study found that even mild fatty liver disease increased a patient’s risk of all-cause death by 71%, and the risk was proportional to the severity of fatty liver disease (3). NAFLD is not only associated with increased liver-related mortality or morbidity but is a multisystem disease affecting multiple extrahepatic organs, including the heart and vascular system. Cardiovascular disease (CAD) is considered in some respects to be an inflammatory disease with an inflammatory pathway similar to NAFLD and is the leading cause of death in NAFLD (4, 5). Visceral adipose tissue promotes eotaxin release from atherosclerotic vascular smooth muscle cells by releasing IL-17, so IL-17 may be a key regulator of local inflammation in CAD lesions and a significant risk factor for CVD (6). According to histological form, NAFLD is divided into simple steatohepatitis and nonalcoholic steatohepatitis (NASH). NASH is a potentially progressive disease that causes cirrhosis in 12-25% of cases within 10 years, significantly increasing the risk of advanced liver disease (7). About 65% of patients with PsA have NAFLD (8). Compared with patients with PsA alone, patients with NAFLD tend to have more severe clinical symptoms, and NAFLD is significantly correlated with Psoriasis (PsO) lesions and disease severity index (PASI). In contrast, PsO, especially PsA, is an important predictor of NAFLD severity. When PsO is present, the likelihood of advanced NAFLD increases by approximately 60%, and progression to NASH is more likely. Furthermore, PsA is even a risk factor for advanced liver fibrosis (9). The pathogenesis of PsA combined with NAFLD is not fully understood, and previous studies have attributed it to a metabolic syndrome caused by factors such as dyslipidemia. Recent studies suggest that chronic low-level systemic inflammation is a common cause of both.

Similar to other autoimmune diseases, a variety of CD4+ T cells and their secreted cytokines are involved in the pathogenesis of PsA. Preliminary studies suggest that PsA is a Th1 cell-mediated disease as Th1 cells and their secretion of interferon-gamma (INF-γ) are significantly increased in skin lesions (10). Likewise, Th17 cells and their secreted cytokine interleukin 17A (IL-17A) are not only present in skin lesions (11), but also have an increased frequency in diseased joints (12). In addition to helper T cells, innate immune cells, especially NK cells, are also closely involved in the pathogenesis of PsA. INF-γ produced by NK cells in the skin is a potent factor for Th17 cell migration into skin lesions. IL-17 producing NK cells are also increased in synovial fluid from patients with undifferentiated spondyloarthropathy closely related to PsA (13).

An increasing number of studies have shown that NAFLD is not a simple liver disease and that the immune system is one of the main drivers of disease progression (14), each stage of NAFLD is accompanied by the accumulation of T cells and NK cells with different functions and phenotypes (15). For example, clinical and animal studies have shown that NAFLD patients have significantly increased liver and peripheral blood Th1 cells and their secreted cytokines IFN-γ, IL-12, and TNF-α (16). In addition, the number of Th17 cells in the liver and peripheral blood of NAFLD patients was also significantly increased (17), which not only mediates liver inflammation and liver injury but also has a significant effect on liver fibrosis (18). NK cells account for 30-55% of the total number of hepatic lymphocytes. Recent studies have found that the number of NK cells in NAFLD patients is reduced (19, 20), while the number of NK cells in the liver of NASH patients is significantly increased (21), which promotes the occurrence and development of liver fibrosis (22, 23), accelerates disease progression and leads to an increased risk of advanced liver disease.

Therefore, PsA and NAFLD share similar underlying inflammatory pathways, and inflammatory cells and pro-inflammatory cytokines associated with PsA may be involved in the occurrence and development of NAFLD through a common chronic inflammatory pathway. However, there is currently no research on the role of inflammatory cells in the pathogenesis of NAFLD and whether they are also involved in abnormal metabolic processes such as lipid disorders. In this study, by looking for the differences in peripheral blood lymphocyte subsets and CD4+T cells in PsA-NAFLD patients, we identified the immunological high-risk factors of PsA patients complicated with NAFLD, development, and assessment of predictive nomograms for NAFLD risk in patients with PsA, and to further explore the correlation between risk factors and dyslipidemia. Early identification and scientific management of risk factors can not only reduce the incidence of NAFLD and the risk of NAFLD-induced advanced liver disease but also effectively control the disease and improve the quality of life of patients.

## Materials and methods

### Clinical date

Our study collected 127 patients with PsA admitted to the Rheumatology Department of the Second Hospital of Shanxi Medical University from August 2016 to June 2021, including 53 males and 74 females, with an average age of 46.5 ± 13.26 years. All patients were diagnosed according to the CASPAR classification diagnostic criteria revised in 2006 (24). The diagnosis of NAFLD mainly relies on liver ultrasound. In addition, 56 healthy controls were recruited from the physical examination center of our hospital. Patients with other autoimmune diseases, serious infections especially viral hepatitis, tumors, and drug-induced hepatitis were excluded. Exclusion criteria also included alcohol consumption > 20 g/day, recreational drug use, and exposure to environments known to induce hepatic steatosis. Clinical and laboratory data, including blood cell analysis, erythrocyte sedimentation rate (ESR), C-reactive protein (CRP), liver enzymes, bilirubin, lipids, and immunoglobulins, were retrospectively collected from patients and healthy controls. All blood samples were collected on an empty stomach on the morning of admission. According to the presence or absence of NAFLD, the patients were divided into two groups, the PsA group (PsA) and PsA combined NAFLD group (PsA-NAFLD). This study was approved by the Ethics Committee of the Second Hospital of Shanxi Medical University (2019YX114). A written informed consent was obtained from every participant.

### Flow cytometry for absolute counts of peripheral blood lymphocytes and CD4+T cell subsets

Whole blood samples from patients with PsA were collected in heparin anticoagulant tubes, and the peripheral blood mononuclear cell suspension was prepared by Ficoll-hypaque density gradient centrifugation. Different antibodies were added in turn for the combined staining of peripheral blood lymphocyte subsets and CD4+T cells. The staining protocol for peripheral blood lymphocyte subsets is as follows: anti-CD3-FITC/CD8-PE/CD45-PercP/CD4-APC antibody is used to label T lymphocytes, anti-CD3-FITC/CD16+56-PE/CD45-PercP/CD19-APC antibody is used to label B lymphocytes and NK cells. CD4+T cell subsets were stained and identified by the following protocols: anti-CD4-FITC/IFN-γ-APC (intracellular staining) for Th1 cells and anti-CD4-FITC/IL-4-PE (intracellular staining) for Th2 Cells, anti-CD4-FITC/IL-17-PE (intracellular staining) to detect Th17 cells, anti-CD4-FITC/CD25-APC/FOXP3-PE (intracellular staining) to identify and detect Treg cells. All immunofluorescent antibodies were purchased from BD Biosciences (BD Biosciences, Franklin Lakes, NJ, USA), blood samples were mixed, incubated, and washed according to the manufacturer’s recommendations, and stained samples were detected within 24 hours using FACSCalibur flow cytometer and BD Multitest software (BD Biosciences, Franklin Lakes, NJ, USA).

### Statistical analysis

Statistical analysis was performed using SPSS software (version 23.0; SPSS Inc., Chicago, IL, USA) and R software (4.1.2, USA). Count data were tested using the chi-square goodness-of-fit test. Measurement data were measured using the Kolmogorov-Smirnov test and Levene t-test; mean ± standard deviation was used to express normality and homogeneity of variance. The independent samples t-test was used for comparison between two groups, and the analysis of variance was used for comparison between groups. Non-normally distributed data are expressed as median (M) and interquartile range and tested using the Kruskal-Wallis H test. Correlation analysis was performed using Pearson’s or Spearman’s correlation. Univariate and multivariate binary logistic regression analysis was used to screen independent risk factors for PsA combined with NAFLD, and a nomogram prediction model was constructed using these risk factors. The discriminative power of the model was evaluated by the area under the receiver operating characteristic curve (AUROC) and the C-index; the calibration of the model was evaluated by the calibration curve and the Hosmer-Lemeshow test; the DCA curve was used to verify the clinical validity of the model.

## Results

### Comparison of demographic and clinical features and laboratory data between the PsA group and PsA-NFFLD group

A total of 127 patients with PsA were included in this study, including 53 male patients and 74 female patients. The mean age of the patients was 46.5 ± 13.26 years. The main demographic characteristics, disease characteristics, and serological parameters of the two groups of patients are shown in **Table 1**. There were no significant differences between the PsA group and the PsA-NAFLD group in terms of sex, age, disease course, and family history. The BMI of PsA-NAFLD patients was significantly higher than that of PsA patients. Diabetes and hypertension are risk factors for NAFLD. The prevalence of hypertension in PsA-NAFLD was 30.43%, which was significantly higher than that in the PsA group (12.35%). In addition, there were no significant differences in medication use between the two groups. The use rate of tumor necrosis factor inhibitor (54.35%) was higher in the PsA-NAFLD group, but the difference was not statistically significant.

**Table 1:** Clinical characteristics of PsA group and PsA-NAFLD group.

	PsA (n=81)	PsA-NAFLD (n=46)	*p*
Demographics			
Age (years)^a^	45.62 ± 13.25	48.07 ± 13.27	0.320
Male n (%)	50 (61.72%)	24 (52.17)	0.389
Female n (%)	31 (38.27%)	22 (47.83%)	
BMI ^a^	22.63 ± 3.83	25.97 ± 4.57	< 0.001
Disease duration (month) ^b^	25.00 (4.00-108.00)	25.00 (6.88-88.50)	0.730
Family history of PsA (%)	18 (22.22%)	10 (21.74%)	1
Risk factors for NAFLD			
Smoking n = (%)	11 (13.58%)	10 (21.74%)	0.347
Drinking n = (%)	7 (8.64%)	6 (13.04%)	0.63
Hypertension n (%)	10 (12.35%)	14 (30.43%)	0.023
Diabetes n (%)	4 (4.94%)	4 (8.7%)	0.647
Current use of medication			
NSAIDs n (%)	69 (85.19%)	38 (82.61%)	0.897
DMARDs n (%)	43 (53.09%)	26 (56.52%)	0.851
Methotrexate n (%)	26 (32.10%)	16 (34.78%)	0.910
Leflunomide n (%)	7 (8.64%)	3 (6.52%)	0.933
Sulfasalazine n (%)	13 (16.05%)	8 (17.39%)	0.998
TNF-α inhibitors n (%)	33 (40.74%)	25 (54.35%)	0.196
Laboratory Characteristics			
ESR (mm/h) ^b^	30.50 (18.00-68.00)	26.50 (14.50-58.50)	0.380
CRP (mg/ml) ^b^	9.65 (3.33-24.63)	11.50 (3.13-26.00)	0.640
Complete blood count			
WBC (*10^9^/L) ^b^	6.14 (4.78-7.17)	6.83 (5.70-8.14)	0.029
Hb (g/L) ^a^	125.00 (116.00-137.50)	135.50 (121.00-151.25)	0.004
PLT (*10^9^/L) ^b^	260.00 (206.50-321.50)	261.00 (211.50-308.00)	0.840
LY (*10^9^/L) ^b^	1.71 (1.37-2.04)	2.01 (1.63-2.20)	0.077
Liver Function Test			
ALT (U/L) ^b^	14.50 (10.65-21.05)	17.95 (12.9-25.60)	0.040
AST (U/L) ^b^	17.5 (14.20-21.50)	17.65 (13.98-23.33)	0.760
GGT (U/L) ^b^	17.80 (12.80-25.40)	24.20 (17.70-45.40)	0.013
TBIL(μmol/L) ^b^	10.50 (8.80-13.50)	12.60 (9.75-16.10)	0.040
TG (mmol/L) ^b^	1.09 (0.81-1.43)	1.47 (1.11-1.81)	< 0.001
CHOL (mmol/L) ^b^	3.82 (3.19-4.68)	4.34 (3.73-4.87)	0.020
HDL (mmol/L) ^b^	1.10 (0.95-1.36)	1.04 (0.86-1.25)	0.090
LDL (mmol/L) ^b^	2.09 (1.75-2.68)	2.56 (2.16-2.94)	0.005
Immunoglobulin			
IgG (g/L) ^b^	12.40 (10.60-14.50)	10.10 (11.70-13.80)	0.150
IgA (g/L) ^b^	2.94 (2.12-3.69)	2.51 (1.99-3.38)	0.140
IgM (g/L) ^b^	0.95 (0.75-1.31)	0.91 (0.73-1.21)	0.095

a Results are expressed as the mean ± standard deviation.

b Results are expressed as the median and 25th and 75th percentiles.

BMI, body mass index; DMARDs, disease-modifying antirheumatic drugs; NSAID, nonsteroidal anti-inflammatory drug; ESR: erythrocyte sedimentation rate; CRP, C-reactive protein; WBC, white blood cells; Hb, Hemoglobin; PLT, platelets; LY, lymphocytes; ALT, Alanine transaminase; AST, Aspartic transaminase; GGT, Gamma-glutamyltransferase; TBIL, Total bilirubin; TG, Triglycerides; CHOL, Cholesterol; LDL, Low-density lipoprotein; HDL, High-density lipoprotein; IgG, Immunoglobulin G; IgA, Immunoglobulin A; IgM, Immunoglobulin M.

By comparing the differences in blood cell analysis between the two groups, it was found that the peripheral blood white blood cell count and hemoglobin content in the PsA group were significantly lower than those in the PsA-NAFLD group. In addition, there were significant differences in liver function-related indicators between the two groups. First, the partial liver enzymes (Alanine transaminase, Gamma-glutamyltransferase) and total bilirubin in the PsA-NAFLD group were significantly higher than those in the PsA group. Since the main diagnosis of NAFLD in this study is based on abdominal ultrasound, it is difficult to accurately determine the degree of NAFLD lesions, so we speculate that the reason for the elevated liver enzymes in PsA-NAFLD patients may be that some patients progress to early NASH. Second, the blood lipid levels in the PsA-NAFLD patients were higher, especially the levels of triglycerides, cholesterol, and low-density lipoprotein were significantly higher than those in the PsA group.

### Peripheral Th1 cells and NK cells were elevated in PsA-NAFLD patients

**Figure 1:**
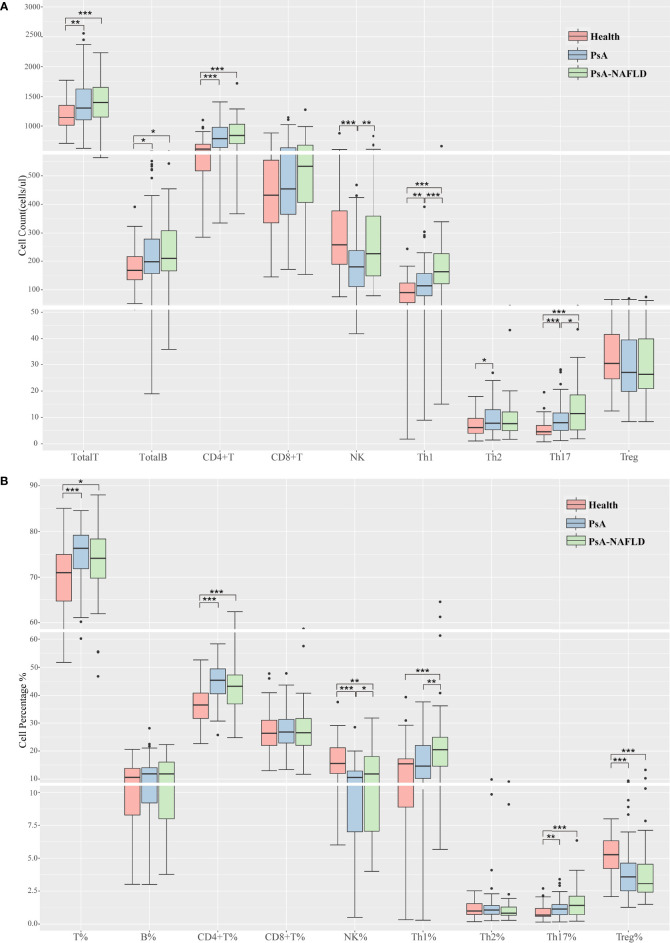
**(A)**. Comparison of peripheral blood lymphocyte subsets and CD4+T cell counts among each study group. **(B)**. Comparison of the proportion of peripheral blood lymphocyte subsets and CD4+ T cells among each study group. (*p < 0.05, **p < 0.01, ***p < 0.001).

We compared the numbers and percentages of peripheral blood lymphocyte subsets and CD4+ T cell subsets between the two groups of PsA patients and healthy controls. Compared with the healthy control group, the number of total T cells (P=0.001) and percentage (P<0.001), the number of total B cells (P=0.050), the number of CD4+T cells (P<0.001), and the percentage (P<0.001), Th1 cell number (P<0.001) Th17 cell number (P<0.001) and percentage (P=0.001), Th17/Treg (P<0.001) all increased in different degrees. The percentage of NK cells (P<0.001) and Treg cells (P<0.001) in the healthy control group were significantly higher than those in the two groups of PsA patients (**Tables 2A, B**, **Figure 1A, B**).

**Table 2:** Absolute counts and proportions of lymphocytes in the peripheral blood in the study participants.

(A)	PsA (n=81)	PsA-NAFLD (n=46)	Health (n=56)	*p*
Total T (cells/μL)	1308.00 1099.51-1659.50	1401.34 1135.48-1675.87	1144.22 1011.73-1357.42	0.001
T%	76.32 71.81-79.38	74.16 69.31-78.49	71.00 64.35-75.17	< 0.001
Total B (cells/μL)	198.11 145.29-278.52	209.50 163.22-323.48	168.00 133.65-220.00	0.050
B%	11.86 9.15-14.14	11.85 7.98-16.27	10.61 8.15-13.91	0.463
NK (cells/μL)	180.00 109.97-238.39	226.01 146.96-371.80	257.00 185.48-377.56	< 0.001
NK%	10.57 6.97-13.19	11.81 7.00-18.68	15.56 12.00-21.29	< 0.001
CD4+T cells (cells/μL)	788.00 640.12-980.00	841.57 694.48-1034.15	613.86 508.65-703.90	< 0.001
CD4+T%	45.40 40.47-49.45	43.28 36.95-47.51	36.63 31.57-41.75	< 0.001
CD8+T cells (cells/μL)	454.16 364.17-639.76	533.21 402.30-687.92	432.11 326.66-555.43	0.090
CD8+T%	26.78 22.65-31.36	26.54 21.88-31.75	26.33 22.00-31.09	0.920
CD4+T/CD8+T	1.72 1.33-2.17	1.62 1.27-1.95	1.39 1.02-1.88	0.039
**(B)**	**PsA (n=81)**	**PsA-NAFLD (n=46)**	**Health (n=56)**	***p***
Th1 (cells/μL)	114.07 79.16-157.78	163.00 119.66-226.69	90.09 51.45-124.76	< 0.001
Th1%	14.63 10.21-22.00	20.48 13.99-25.07	15.44 8.65-17.56	0.001
Th2 (cells/μL)	7.81 5.29-13.02	7.65 4.81-12.18	6.17 3.79-9.68	0.040
Th2%	1.06 0.73-1.42	0.82 0.65-1.33	0.99 0.72-1.55	0.538
Th17 (cells/μL)	7.93 4.86-11.74	11.40 5.10-19.24	4.56 3.42-7.15	< 0.001
Th17%	1.13 0.66-1.47	1.41 0.68-2.11	0.68 0.52-1.21	0.001
Treg (cells/μL)	27.02 19.76-39.96	26.31 20.66-40.73	30.46 24.43-41.79	0.354
Treg%	3.60 2.48-4.67	3.06 2.35-4.60	5.28 4.20-6.35	< 0.001
Th1/Th2	13.90 8.25-23.95	19.79 12.26-31.22	12.74 7.78-19.96	0.011
Th17/Treg	0.29 0.19-0.46	0.47 0.18-0.73	0.14 0.10-0.25	< 0.001

T, T lymphocyte; B, B lymphocyte; NK, natural killer cell.

Th1, T-helper 1 cells; Th2, T-helper 2 cells; Th17, T-helper17 cells; Treg, regulatory T cells.

In addition, we compared the number and percentage of peripheral blood lymphocyte subsets and CD4+T cell subsets in the two PsA groups and found that the number (P<0.001) and percentage (P=0.001) of peripheral blood Th1 cells, Th17 cells (P=0.037), Th1/Th2 ratio (P=0.021), NK cell numbers (P=0.007) and percentages (P=0.041) were significantly increased in the PsA-NAFLD group (**Tables 2A, B**, **Figures 1A, B**).

### Development of the nomogram for prediction of nonalcoholic fatty liver disease in psoriatic arthritis

**Figure 2:**
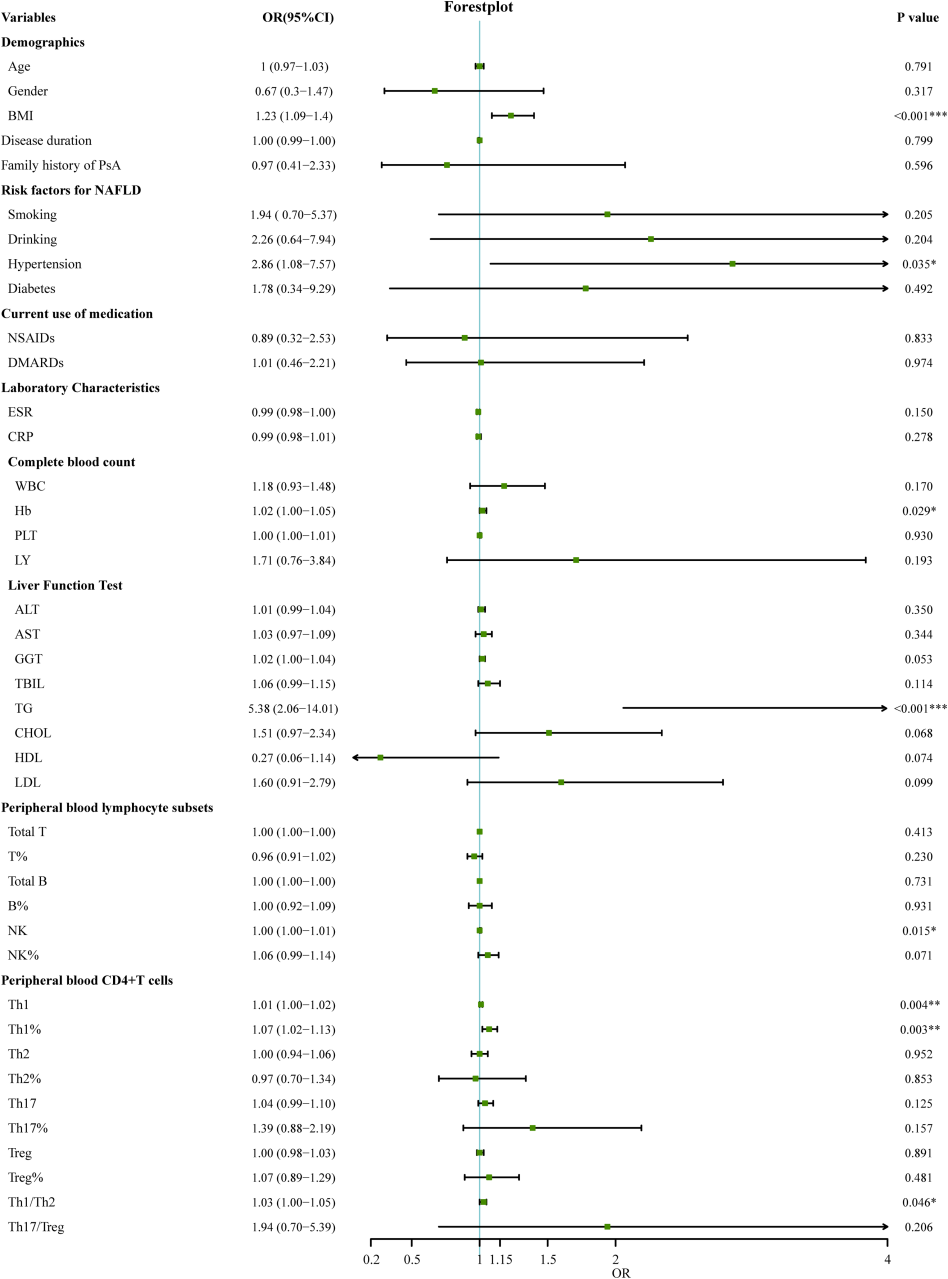
Univariate logistic regression analyses for factors associated with the presence of NAFLD in PsA patients. (*p < 0.05, **p < 0.01, ***p < 0.001). OR, odds ratio; 95%CI:95% confidence interval; BMI, Body Mass Index; DMARDs, disease-modifying antirheumatic drugs; NSAID, nonsteroidal anti-inflammatory drug; ESR, erythrocyte sedimentation rate; CRP, C-reactive protein; WBC, white blood cells; Hb, Hemoglobin; PLT, platelets; LY, lymphocytes; ALT, Alanine transaminase; AST, Aspartic transaminase; GGT, Gamma-glutamyltransferase; TBIL, Total bilirubin; TG, Triglycerides; CHOL, Cholesterol; LDL, Low-density lipoprotein; HDL, High-density lipoprotein; T, T lymphocyte; B, B lymphocyte; NK, natural killer cell; Th1, T-helper 1 cells; Th2, T-helper 2 cells; Th17, T-helper17 cells; Treg, regulatory T cells.

**Figure 3:**
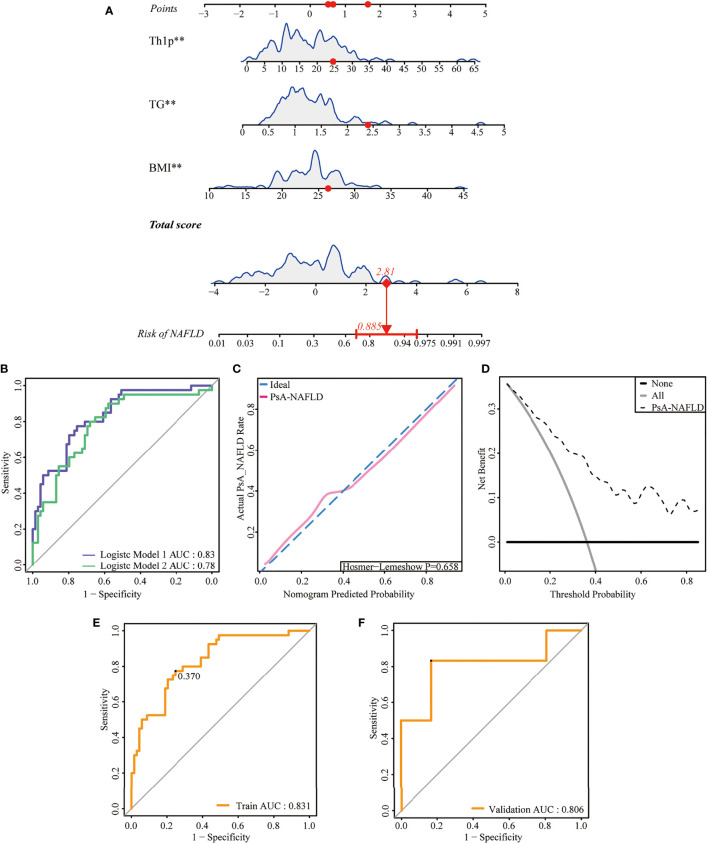
Development and assessment of a nomogram for prediction of NAFLD in PsA. **(A)**. Example of prediction nomogram for risk of NAFLD in PsA patients. The nomogram incorporates the risk factors of Th1%, TG, and BMI. **(B)**. The receiver operating characteristic (ROC) curve for the discrimination of the nomogram to predict the risk of NAFLD in PsA patients. Model 1: The nomogram incorporating the risk factors of Th1%, TG, and BMI; Model 2: The nomogram incorporating the risk factors of TG and BMI. **(C)**. Calibration curve for predicting the risk of NAFLD in PsA patients. The red line along the dashed line indicates that the predicted prevalence is close to the actual prevalence. **(D)**. Decision curve analysis for predicting the risk of NAFLD in PsA patients. **(E)** The receiver operating characteristic (ROC) curve for the discrimination of the nomogram to predict the risk of NAFLD in modeling population. **(F)** The receiver operating characteristic (ROC) curve for the discrimination of the nomogram to predict the risk of NAFLD in validation population **p < 0.01.

The 127 patients with PsA included in this study were divided into the modeling population (102 cases) and the validation population (25 cases) according to the ratio of 8:2 by the random number table method. The auxiliary examination results and other data were compared (P>0.05), the difference was not statistically significant, and the two groups were comparable. Univariate Logistic regression was used to screen the risk factors of NAFLD in PsA patients. The clinical indicators with the statistical difference (P<0.05) are shown in **Figure 2**. Risk factors with P<0.01 in the univariate logistic regression were included in the multivariate logistic stepwise regression analysis. The results showed that in addition to traditional risk factors such as BMI and TG, peripheral blood Th1% was also an independent risk factor for NAFLD in patients with PsA (**Table 3**). The above three independent risk factors were incorporated into the prediction model, and the individualized nomogram prediction model of PsA complicated with NAFLD was established (**Figure 3A**). Considering that peripheral blood Th1% is not a traditional risk factor for NAFLD, we removed Th1% from the above prediction model, re-established a new prediction model 2, and compared the test performance of the two models (**Table 3**).

**Table 3:** Univariate logistic regression analyses for factors associated with the presence of NAFLD in PsA patients.

	Model1		Model2	
	Odds Ratio (95% CI)	*P*	Odds Ratio (95% CI)	*P*
BMI	1.22 (1.07-1.42)	0.005**	1.18 (1.05-1.35)	0.008**
TG	4.78 (1.84-14.41)	0.003**	4.08 (1.66-11.91)	0.004**
Th1%	1.12 (1.04-1.19)	0.001**	–	–
AUC	0.83 (0.76-0.90)		0.78 (0.70-0.87)	
C-Index	0.83		0.79	

BMI, Body Mass Index; TG, Triglycerides; Th1, T-helper 1 cells; AUC, Area Under the ROC Curve. (**p < 0.01).

### Validation of the nomogram

First, we compared the discriminability between the two models. The AUC of Model 1 was 0.83 (95%CI: 0.76~0.90), and the C-index was 0.83. Model 2 had an AUC of 0.79 (95% CI: 0.70–0.87) and a C-index of 0.79, which was worse than the aforementioned models (**Table 3**, **Figure 3B**). This suggests that peripheral blood Th1% in PsA patients plays an important role in predictive models of NAFLD onset.

The discriminability of the model was verified by plotting the ROC curves of the two groups of people. The AUC of the modeling population was 0.83 (95% CI: 0.76-0.90), the C-index was 0.83 (**Figure 3E**), and the AUC of the validation population was 0.81 (95% CI: 0.57-0.87), and the C-index was 0.81 (**Figure 3F**), the C-index of the prediction model in both populations is > 0.75, and the model has good discriminative power.

Next, we plotted the calibration curve of the diagnostic model. The prediction curve of the model is close to the actual observation curve, indicating that the calibration ability of the model is good. The Hosmer-Lemeshow test was further performed on the diagnostic model, and its P-value was 0.658 (P>0.05). The difference was not statistically significant (**Figure 3C**). Furthermore, by plotting the clinical decision curve (DCA), it was shown that the Cutoff value (37.0%) obtained by the ROC analysis (**Figure 3E**) was within the threshold probability range of the DCA curve. Further analysis showed that when 37.0% was set as the threshold probability of diagnosing PsA with NAFLD, 21 out of 100 PsA patients at risk for NAFLD diagnosed using this model could benefit from it without harming others (**Figure 3D**). Therefore, the nomogram of the prediction model has a better prediction effect on the risk of NAFLD in PsA patients and has a certain clinical application value.

### Peripheral blood NK cell levels are associated with dyslipidemia in patients With PsA

**Figure 4:**
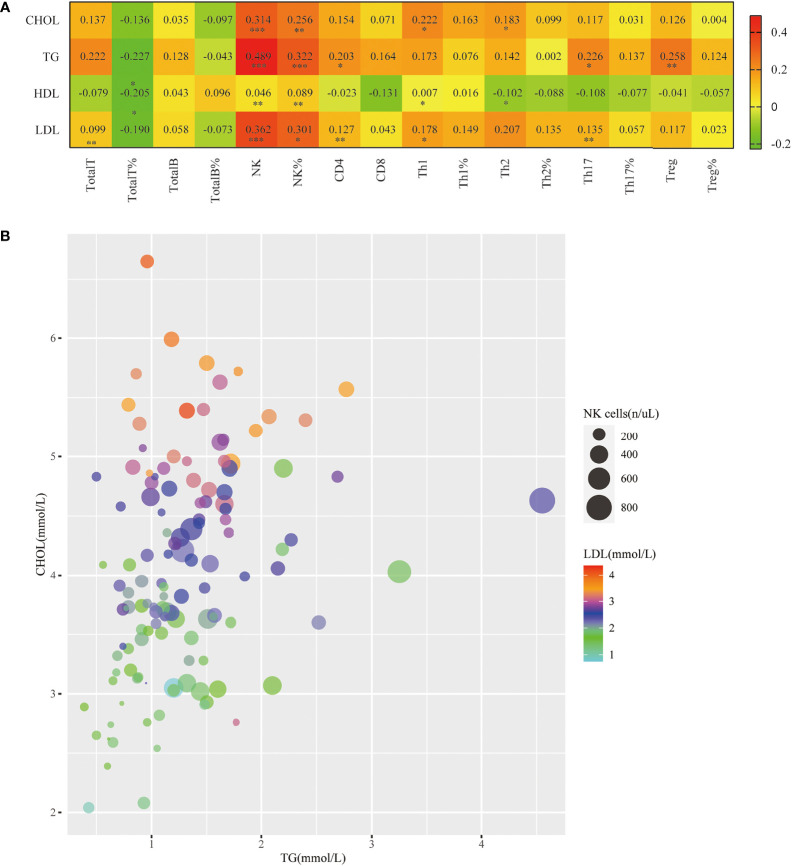
Correlation of peripheral blood NK cell levels with hyperlipidemia in patients with PsA. **(A)**. Heatmap of correlation of the serum lipid levels (TG, CHOL, LDL, HDL) with Total T, Total T%, Total B, Total B%, NK, NK%, CD4+T, CD8+T, Th1, Th1%, Th2, Th2%, Th17, Th17%, Treg, Treg%. (*p < 0.05, **p < 0.01, ***p < 0.001; by *Spearman correlation test*). **(B)**. Correlation bubble plot of TG, CHOL, LDL, and peripheral blood NK cell count.

Dyslipidemia is a traditional risk factor for NAFLD. Our study also showed that the levels of some blood lipids (TG, CHOL, LDL) were significantly higher in the PsA-NAFLD group than in the PsA group. Further analysis of the correlation between common lipids in serum of PsA patients and peripheral blood lymphocyte subsets revealed that the number and percentage of peripheral blood NK cells were positively correlated with CHOL, TG, and LDL levels. Among them, the correlation between the number of NK cells and the level of TG is more obvious (**Figures 4A, B**). This suggests that NK cells may be involved in the occurrence of dyslipidemia in PsA patients.

In addition, we also found that the numbers of Th1 cells, Th2 cells, Th17 cells, and Treg cells in the peripheral blood of PsA patients were all correlated with certain blood lipids, but the correlation was weak (**Figure 4A**).

## Discussion

Previous research on PsA has focused on the skin and joints. In the past ten years, with the in-depth study of the comorbidities of PsA patients, the pathophysiological relationship between PsA and cardiovascular disease, metabolic syndrome, insulin resistance, and mental illness has gradually been recognized. The occurrence of PsA-related comorbidities and their impact on mortality have received increasing attention from scholars (9, 25). Many recent studies have shifted the focus of PsA complications to the liver, especially NAFLD (26, 27). Because NAFLD is not only highly prevalent in patients with PsA, but more importantly, when PsA and NAFLD co-occur, the severity of both diseases may increase significantly. Therefore, early identification and intervention of NAFLD in PsA patients are particularly important.

This retrospective study analyzed the characteristics of peripheral blood lymphocyte subsets and CD4+T cells in patients with PsA and PsA-NAFLD and used univariate and multivariate Logistic regression to find high-risk immunological factors for NAFLD. The results of the analysis showed that Th1% was an independent risk factor for NAFLD. Combined with other risk factors BMI and TG, a nomogram prediction model of NAFLD risk in PsA patients was established and validated, hoping to predict and identify NAFLD early. Furthermore, the relationship between lymphocyte subsets in peripheral blood and serum lipid profile was further discussed to seek the correlation between immune cells and lipid metabolism abnormalities.

PsA is a chronic inflammatory arthritis associated with PsO. Many studies have shown that both PsA and PsO are chronic inflammations mediated by abnormal T cells, and various pro-inflammatory cytokines play key roles in their development (28). Interactions between T-cell subsets, stromal cells, and cytokines in the local microenvironment determine disease characteristics, including colitis, synovitis, bone and cartilage loss, and new bone formation in the axial and peripheral musculoskeletal systems (29). PsA was originally thought to be a Th1 cell-mediated disease. Since the level of Th1 cells and their secreted INF-γ in skin lesions was significantly increased, *in vitro* experiments showed that IFN-γ could enhance the proliferation of keratinocytes (30). And in successfully treated PsO patients, the levels of Th1 cells in skin lesions and peripheral blood were significantly reduced (31). Recent studies suggest that Th17 cells and their secreted IL-23/IL-17 play a central role in the pathogenesis of PsA, similar to other autoimmune diseases (32). The levels of Th17 cells and IL-17 in the skin lesions of PsA patients were much higher than those in healthy skin, especially IL-17A played a key role in maintaining PsO plaque inflammation, and IL-17A mRNA levels correlated with disease activity. Th17 cells and IL-17 levels are also elevated in the synovial fluid of patients with PsA (33). Randomized clinical trials show that IL-17A and IL-17F antibodies have good efficacy and are potential therapeutic targets (34). Notably, these two types of CD4+T cells promote and activate each other during the pathogenesis of PsA. IFN-γ secreted by Th1 cells can induce Th17 cells through IL-1 and IL-23, and the overactivity of Th17 cells can lead to the exacerbation of Th1 immune responses and the development of chronic inflammatory states (33). Thus, Th1 cells and Th17 cells play a central role in systemic chronic inflammation in PsA. Our study also showed that the levels of Th1 cells and Th17 in the peripheral blood of PsA patients were significantly higher than those of healthy people, suggesting that their abnormal levels may be involved in the pathogenesis of PsA.

Treg cells, a subset of CD4+T lymphocytes, can suppress effector T cells and inflammation, and play an important role in maintaining autoimmune tolerance. Existing studies on the level of Treg cells in the lesional skin and peripheral blood of PsO are still controversial, but most studies have shown that the number and function of Treg cells in peripheral blood are defective (35), and the ratio of Th17/Treg was significantly positively correlated with PASI score (36). Our study also demonstrated decreased Treg% and Th17/Treg cell imbalance in peripheral blood of PsA patients.

In addition to CD4+T cells, NK cells also play a role in the skin lesions of PsA. Studies have shown that cellular infiltrates in acute psoriatic plaques include 5-8% NK cells and are primarily confined to the middle and papillary dermis. NK cells also function by secreting cytokines such as INF-γ, TNF, and IL-22. Once NK cells enter the diseased skin, they will produce INF-γ, which is an effective factor for Th17 cells to migrate to the skin lesions (37). It is unclear whether peripheral blood NK cells are involved in the pathogenesis of PsO, but multiple studies have shown that peripheral blood NK cells are reduced in PsO patients. Our findings also showed that both the number and percentage of NK in the peripheral blood of patients with PsA decreased.

Notably, chronic systemic inflammation caused by abnormal levels of CD4+T cells and NK cells in PsA patients promotes the occurrence and development of hepatic steatosis, especially NAFLD, and significantly increases the risk of advanced liver disease. Because the two diseases share a common inflammatory pathway, which plays a bidirectional promoting role in the course of both diseases. Therefore, identifying key links in the inflammatory pathway and intervening early can not only prevent the occurrence of NAFLD but also help control the disease of PsA. Our study showed that the level of CD4+T cells in peripheral blood of PsA-NAFLD patients was significantly different from that of PsA patients, especially the number and percentage of Th1 cells, and the number of Th17 cells and the ratio of Th1/Th2 were significantly increased. In addition, the number and percentage of NK cells also showed a significant upward trend.

CD4+T cells are involved in the pathological process of NAFLD and promote the occurrence of liver fibrosis and even liver cancer. A recent study explored the role of CD4+T cells in a high-fat diet-induced (HFD) mouse model of humanized NAFLD and found that it has an important role in promoting liver fibrosis. In HFD mice, marked infiltration of CD4+T cells was found and the levels of proinflammatory cytokines IFN-γ, IL-6, IL-17A, and IL-18 were elevated. However, depletion of CD4+T cells in HFD mice not only prevented liver steatosis but also significantly reduced immune infiltration and liver fibrosis (38). Th1 cells, as an important part of CD4+T cells, play an important role in the pathogenesis of NAFLD. Levels of hepatic Th1 cells and related cytokines IFN-γ, IL-12, and TNF-α are elevated in steatotic mice on a choline-deficient diet, a process associated with increased STAT4 and T-bet expression (39). In addition, elevated Th1 cells may be involved in the development of liver fibrosis in the advanced stages of the disease. Human studies also found elevated levels of Th1 cells in peripheral blood and liver in NAFLD patients, with no significant difference between NAFLD and NASH (16).

It has been confirmed that Th1 cells are involved in the pathological process of obesity-related adipose tissue inflammation. Th1 cells are abundant in subcutaneous and visceral adipose tissue in an HFD-fed obese mouse model (40, 41), and blocking Th1 function can reduce adipose tissue inflammation and alleviate symptoms of impaired glucose tolerance (42). Furthermore, in morbidly obese adult patients, genes involved in T cell activation and Th1 cell phenotype switching were already upregulated in the liver before the characteristic leukocyte infiltration occurred (43), this series of evidence suggests that Th1 cells may be involved in the early stages of NAFLD. Through multivariate logistic regression analysis, our study found that peripheral blood Th1% in PsA patients was an independent risk factor for NAFLD. Combined with other risk factors BMI and TG, a nomogram prediction model of NAFLD risk in PsA patients was constructed. Considering that Th1% is not a traditional risk factor for NAFLD, a new predictive model was reconstructed after removing Th1% from the model. By plotting the ROC curve, it is found that the discriminative power of the new model is worse than the original model, so Th1% plays an important role in the prediction model. When we used the cutoff value of the ROC curve of the prediction model (37.0%) as the threshold of the DCA curve, the clinical net benefit rate of patients was higher than the two extreme methods of no intervention and all intervention. This suggests that patients will benefit from immediate intervention when the model predicts risk of NADLD higher than 37.0%, and if the risk is lower than 37.0%, the intervention is temporarily withheld. Therefore, this model is beneficial to the formulation of clinical decision-making programs for PsA patients.

The role of Th17 cells in NAFLD has been extensively studied, and relevant animal and human studies have shown that Th17 cells are elevated in peripheral blood and liver tissue (14), especially in NASH patients (44). IL-17 secreted by Th17 cells is an important factor in promoting hepatic steatosis. Numerous animal and *in vitro* studies have found that hepatic steatosis increases when IL-17 is present but decreases after blocking IL-17 function (39, 45). In addition, Th17 cells also have obvious effects on liver fibrosis, and the possible mechanism is that IL-17 directly acts on hepatic stellate cells in a JNK- and STAT3-dependent manner to induce collagen production (46).

To date, the level and function of NK cells in NAFLD remain controversial. Recent studies have shown that NAFLD patients have a lower frequency of CD56dim NK cells and lower expression of the activating receptor NKG2D compared to healthy individuals (22). Furthermore, NK cell function is impaired, resulting in reduced granzyme/perforin and INF-γ production, ultimately reducing cytotoxicity and tumor lethality (19). However, in NASH patients, the level of NK cells in the liver parenchyma is increased, which directly activates hepatic stellate cells through the activation of receptors NKG2D, NKp46, and p38/PI3K/AKT pathways, and promotes the development of liver fibrosis (23, 47, 48). Our study also showed that the percentage of peripheral blood NK in PsA-NAFLD patients was lower than that in healthy people, but it is worth noting that the number and percentage of peripheral blood NK increased in the PsA-NAFLD group compared with the PsA group. We speculate that it may be associated with higher levels of systemic inflammation in patients with PsA-NAFLD. Considering the role of NK cells in NASH and liver fibrosis, the occurrence of NASH and advanced liver disease in PsA-NAFLD patients should be monitored in clinical practice.

Dyslipidemia is a common risk factor for both diseases, in addition to the common chronic low-grade systemic inflammation. PsA patients are often accompanied by a variety of lipid metabolism abnormalities, such as increased TG and decreased High-density lipoprotein cholesterol (HDL-C), which are more prevalent during disease activity (49), suggesting a potential link between inflammation and lipid profiles. Abnormal lipid metabolism is also closely associated with NAFLD, manifested by increased levels of TG and low-density lipoprotein cholesterol (LDL-C) and decreased high-density lipoprotein cholesterol (HDL-C) levels (50), therefore PsA and NAFLD have similar lipid profiles. However, the lipid profile of PsA-NAFLD patients has not been reported. In our study, serum TG, CHOL, and LDL levels were significantly higher in the PsA-NAFLD group than in the PsA group. It is worth noting that the number and percentage of NK cells in peripheral blood were positively correlated with these three lipids, especially the correlation between the number of NK cells and TG was more significant. At present, the research on the relationship between NK cells and lipid metabolism such as TG is still relatively limited. Recent studies have found that in the NASH mouse model, liver NK cells are significantly increased, and after NK cell depletion, liver TG content is reduced, and the symptoms of hepatitis in mice are significantly relieved, suggesting that NK cells are involved in the process of liver lipid accumulation (51). Therefore, given the correlation of NK cell levels with differential lipids, we speculate that NK cells may be involved in the occurrence of dyslipidemia in PsA patients, thereby increasing the susceptibility to NAFLD. The exact relationship between peripheral blood NK cells and dyslipidemia in PsA patients still requires epidemiological investigations with large samples.

Of course, our study has certain limitations. First, the modeling data came from the same medical center and lacked validation from external data. If patient data from multiple centers can be incorporated at a later stage, with simultaneous external validation, the model can be further adjusted and strengthened. Second, the independent risk factors in the nomogram prediction model are all continuous measurement variables, which are not clinically concise. In the later stage, the method of optimal scale regression can be considered to group the continuous measurement data variables and convert them into categorical variables, which is convenient for the clinical application of the nomogram prediction model. Finally, because this study was a retrospective clinical analysis, an accurate assessment of PsA disease activity and NAFLD severity was lacking.

In conclusion, NAFLD is a common complication of PsA. If not detected and intervened early, it may lead to the progression of PsA and even increase the risk of advanced liver disease. Our study found that Th1% in peripheral blood of PsA patients was an independent risk factor for NAFLD. Combined with the traditional risk factors of hyperlipidemia and obesity, an individualized nomogram prediction model was constructed to predict the risk of NAFLD in PsA at an early stage. The prediction model has good discrimination, calibration, and clinical validity, and the visualized prediction model is convenient for clinical operation. In addition, the level of NK cells in peripheral blood is related to dyslipidemia in patients with PsA, and may also be involved in the pathogenesis of NAFLD. Therefore, Th1 cells and NK cells may be potential biomarkers to detect NAFLD in PsA patients. In the future, we will analyze the levels of inflammatory cytokines downstream of Th1 and NK cells to explore their molecular biological mechanisms.

## Data availability statement

The raw data supporting the conclusions of this article will be made available by the authors, without undue reservation.

## Ethics statement

This study was approved by the Ethics Committee of the Second Hospital of Shanxi Medical University (2019YX114). A written informed consent was obtained from every participant.

## Author contributions

Individual authors’ contributions are as follows: BL performed the data analyses and wrote the manuscript. HY and JL participated in the collection of samples and clinical data. RS participated in the performance of the research and statistical analysis. CG and XL participated in the study design and revising of the manuscript. CW provided intellectual input and supervision throughout the study and made a substantial contribution to manuscript drafting. All authors contributed to the article and approved the submitted version.

## Funding

This study was funded by the National Natural Science Foundation of China (No. 81971543), National Natural Science Foundation of China (No. 81471618), and Key Research and Development (R&D) Projects of Shanxi Province (201803D31119).

## Conflict of interest

The authors declare that the research was conducted in the absence of any commercial or financial relationships that could be construed as a potential conflict of interest.

## Publisher’s note

All claims expressed in this article are solely those of the authors and do not necessarily represent those of their affiliated organizations, or those of the publisher, the editors and the reviewers. Any product that may be evaluated in this article, or claim that may be made by its manufacturer, is not guaranteed or endorsed by the publisher.
